# Arrhythmic episodes in patients implanted with a cardioverter-defibrillator – results from the Prospective Study on Predictive Quality with Preferencing PainFree ATP therapies (4P)

**DOI:** 10.1186/s12872-019-1121-4

**Published:** 2019-06-17

**Authors:** François Regoli, Denis Graf, Beat Schaer, Firat Duru, Peter Ammann, Lorenza Mangoni di S. Stefano, Barbara Naegli, Haran Burri, Rainer Zbinden, Nazmi Krasniqi, Martin Fromer, 4P Study Group, Tiziano Moccetti, Angelo Steffel, Jan Steffel, Urs Eriksson

**Affiliations:** 1 Department of Cardiology, Fondazione Cardiocentro Ticino, Via Tesserete 48, 6900 Lugano, Switzerland; 2 Cantonal Hospital of Fribourg (HFR), Fribourg, Switzerland; 3 University Hospital of Basel (KSB), Basel, Switzerland; 4 University Hospital of Zurich (USZ), Zürich, Switzerland; 5 Cantonal Hospital of St. Gallen (KSSG), St. Gallen, Switzerland; 6 Medtronic Core Clinical Solutions, Rome, Italy; 7 Klinik im Park, Zurich, Switzerland; 8 University Hospital of Geneva (HUG), Geneva, Switzerland; 9 Triemli Hospital, Zurich, Switzerland; 10 GZO Hospital, Wetzikon, Switzerland; 11 University Hospital of Lausanne (CHUV), Lausanne, Switzerland

**Keywords:** Ventricular tachyarrhythmia, Implantable cardioverter-defibrillator, Antitachycardia pacing, Shock

## Abstract

**Background:**

Little is known about the ICD performance using enhanced detection algorithms in unselected, non-trial patients. Performance of recent generation ICD equipped with SmartShock™ technology (SST) for detection and conversion of ventricular tachyarrhythmias (VTA) was investigated.

**Methods:**

4P was a prospective, multicenter, observational study conducted in 10 Swiss implanting centers. Patients with a Class I indication according to international guidelines were included and received an ICD with SST. ICD discrimination capability was assessed by evaluating SST performance; therapy efficacy was assessed by rate of VTA conversions by ATP and by rescue shocks.

**Results:**

Overall, 196 patients were included in the analysis with a mean duration of follow-up of 27.7 months (452 patient-years of observation). Patient-specific rather than recommended programming was preferred. Device-detected episodes were frequent (5147 episodes in 146 patients, 74.5%). In 44 patients (22.4%), 1274 episodes were categorized as VTA; only 215 episodes were symptomatic. ATP was the first-line therapy and highly effective (99.9% success rate at the episode level, 100.0% at the patient level). Rescue shocks were rare (66 episodes in 28 patients); 7 shocks in 5 patients (2.6%) were inappropriate. Death and hospitalization rates were low.

**Conclusions:**

In a cohort of non-trial, unselected ICD patients, VTA episodes were frequent. The 4P results confirm the robustness of VTA detection by SST and the effectiveness of ATP treatment, hence limiting overall ICD shock burden.

## Background

Implantable cardioverter defibrillator (ICD) therapy is the mainstay for the primary and secondary prevention of arrhythmic sudden death by treating ventricular tachyarrhythmias (VTA) in three programmable rate zones: ventricular tachycardia (VT), fast ventricular tachycardia (FVT), and ventricular fibrillation (VF) [1]. The indications for ICD implantation are based on solid results from many randomized controlled endpoint trials in carefully selected patient populations. However, in daily practice, patients may differ substantially from those included in pivotal trials. This raises the question of whether the benefits seen in a controlled trial would also apply under the more heterogeneous conditions of daily practice in terms of such clinical outcomes as hospitalizations, mortality, and clinically symptomatic events.

By far, the leading cause of inappropriate therapy is the misclassification of supraventricular tachycardia (SVT), most commonly atrial fibrillation with high ventricular rates. Other causes include intracardiac (T-wave) oversensing, extra-cardiac (lead) noise, and non-sustained or self-terminating VT/VF [2, 3]. As a consequence, evidence-based shock reduction strategies based on enhanced detection algorithms, such as SmartShock™ technology (SST), have been developed and investigated in well-designed clinical trials [4].

Latest generation ICDs are antitachycardia-pacing (ATP) devices with defibrillation backup [5, 6]. Ultimately, better discrimination has led to improved ATP efficacy thus reducing overall ICD shocks and their deleterious effects [7–9].

The aim of the *Prospective Study on Predictive Quality with Preferencing PainFree ATP therapies* (4P) was to generate real-world evidence on technical and clinical outcomes of detected, categorized and treated arrhythmic episodes occurring in patients who received an ICD with SST enhanced detection algorithms.

## Methods

### Study design

The Prospective Study on Predictive Quality with Preferencing PainFree ATP therapies (4P) was a prospective, multicenter, observational study of planned 24 months duration aimed at generating real-world evidence of ventricular tachyarrhythmia management by implantable cardioverter defibrillators (ICD) under conditions of daily practice. The study is registered on clinicaltrials.gov with the reference number NCT01509378.

### Study population

Eligible patients were adults who gave their written informed consent for participation in the study and implantation of an ICD based on a Class I indication for primary or secondary prevention of sudden cardiac death according to the latest published guidelines [10, 11]. Single-chamber (SC), dual-chamber (DC) and triple-chamber (CRT-D) devices, either as new implants, upgrades or replacements, were included.

Leads could be from any manufacturer. A connection to the CareLink™ network was required. Patients with permanent atrial fibrillation, a life expectancy of less than 24 months due to another non-cardiac disease or participating in another concomitant trial were excluded. The study was conducted in accordance with the Declaration of Helsinki. Ethical Review Board approval was obtained prior to study start from the corresponding institutions of all participating centers.

### Study outcomes of interest

The primary objective of the study was to document device activity and performance in patients with a Class I indication seen in daily practice. In order to assess SST capability to detect and categorize device-based episodes, sensitivity, specificity, positive- and negative predictive values were computed based on the number device-detected, discriminated and categorized VTAs, ATP therapies, and rescue shocks. Computation of episodes considered both number of episodes and number of patients having experienced one or more of such episodes during the follow-up period. The secondary objective was to report medical outcomes in these patients: adjudicated symptomatic events; hospitalizations (all-cause, cardiac, and arrhythmia-related), deaths (all-cause, cardiac), and severe adverse events including serious adverse device effects (SADE). Finally, in addition to symptoms registered at any of the follow-up visits, patients were encouraged to consult or to inform the follow-up center if they had symptoms suggestive of arrhythmia episodes such as syncope, pre-syncope, palpitations or shock. An expert board of two experienced investigators and one external expert, not involved in the trial, analyzed these events. Symptomatic events were adjudicated and classified as VTA or not by the expert board, using the device-recorded data.

### Device features and programming

All implanted ICD devices featured enhanced detection algorithms integrated in the SST (Medtronic Inc., Minneapolis, MN, USA) package, which includes: *Lead Noise Discrimination* differentiates RV lead noise from VT/VF by comparing a far-field EGM signal to near-field sensing; *RV Lead Integrity Alert* extends the VF detection time, triggers programmable alerts and increases diagnostic data collection and monitoring in case of lead malfunction; *PR-Logic* and *Wavelet* are algorithms that differentiate ventricular from supraventricular rhythms considering either the relation between A and V EGMs, for PR-logic, or morphology of the V EGM during tachycardia compared to V EGM during sinus rhythm; *T-wave oversensing* withholds therapy if there is evidence that a fast ventricular rate results from double-counting due to T-wave oversensing; *Confirmation +* confirms the presence of an arrhythmia by comparing the rhythm cycle length to a calculated confirmation interval before a shock is delivered following the capacitor charge.

All participating centers received a recommendation for ICD programming strategies based on available evidence. Such evidence-based programming was recommended for all patients. In brief, in primary prevention, recommended cycle length for VF detection was 320 ms with an initial Number of Intervals to Detect (NID) of 30/40 [12]. In addition, FVT and VT detection was to be set OFF. The VT monitoring zone was based on a cycle length of 400 ms and a NID of 32 [12]. All SST algorithms were to be switched ON with SVT limit set at 260 ms [13–15]. In secondary prevention, the same settings applied except the recommended cycle length for VF detection was 300 ms with initial NID 30/40 [16] and VT detection switched ON with a cycle length of 360 ms with an initial NID of 16. Primary therapy was ATP [11]. These programming settings are based on published scientific evidence [11, 13–16] and were recommended as such in the study protocol. Accordingly, specific programming information was handed out to each implanter as part of the study documentation before trial initiation (Figs. 5 and 6 in Appendix). The protocol allowed divergences based on the judgment of the implanting or follow-up physician.

### Statistical analysis

All patients who matched with the inclusion and exclusion criteria were included in the statistical analysis. Descriptive statistics were used for baseline characteristics and outcomes of interest. No imputation was done for missing data. Exploratory significance testing was performed between the three device groups (SC-ICD, DC-ICD, CRT-D), a two-sided *p* value of less than 0.05 being required for significance. The Bonferroni correction was applied for the post-hoc comparisons. Odds Ratios (OR) with 95% confidence intervals (95% CI) were calculated using univariate logistic regression methods for identifying predictors of technical and medical outcomes of interest. SST performance was assessed by computing sensitivity, specificity, as well as negative and positive predictive values. Time to first medical outcomes (hospitalizations, deaths) was described by Kaplan-Meier curves with Cox regression models applied for adjustment between study centres and the Hazard Ratios (HR) with 95% CI were reported. The annual rates of device therapies and clinical events were presented per 100 patient years together with the Poisson 95% CI. Treatment success was defined as the absence of VTA redetection following therapy delivery. The power calculation was based on the results in the ATP arm of the PainFree Rx II trial [11]. This trial compared the efficacy of ATP for shock prevention in 313 patients with 4230 spontaneous episodes during a mean follow-up time of 11 months. Using a translational approach, 200 patients followed during 24 months were deemed necessary to be included in the 4P study. All analyses were performed with the SAS 9.4 software package (SAS Institute Inc., Cary, NC, USA) by a senior statistician expert in the field (L.M.).

## Results

### Study population

**Fig. 1:**
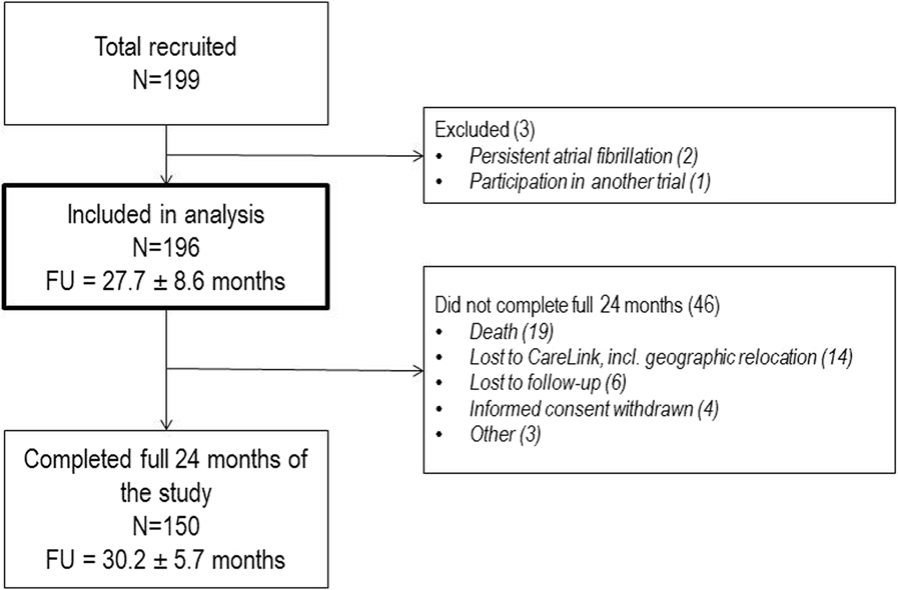
Patient-flow diagram

Between September 2011 and January 2014, 199 patients were enrolled in 10 participating centers in Switzerland. Three patients were protocol violators and excluded from the analysis, two of them because of permanent atrial fibrillation at baseline and one because of participation in another trial. Thus, 196 patients in three groups (48 SC-ICD, 50 DC-ICD and 98 CRT-D) were included in the analysis (Fig. 1). Detailed baseline characteristics presented in Table 1 show that CRT-D patients were older, more clinically compromised with significantly lower left ventricular ejection fraction and a significantly higher proportion of patients with symptomatic heart failure in NYHA II-IV compared to the other 2 groups.

**Table 1:** Baseline characteristics in the 4P-study

	SC-ICD (*N* = 48)	DC-ICD (*N* = 50)	CRT-D (*N* = 98)	All patients (*N* = 196)	*p* value
Patient demographics and clinical presentation					
Male (%)	81	88	84	84	0.645
Age (years ± SD)	59 ± 13	63 ± 14	68 ± 11	64 ± 13	0.001^2^
LVEF (% ± SD)	44 ± 14	42 ± 14	31 ± 10	36 ± 13	< 0.001^1,2^
QRS (ms ± SD)	107 ± 26	112 ± 30	143 ± 35	126 ± 36	< 0.001^1,2^
Secondary prevention (%)	58	48	17	35	< 0.001^1,2^
Device replacement or upgrade (%)	63	41	61	56	0.039
NYHA functional class (%)					
I	17	18	10	14	< 0.001^1,2^
II	19	24	50	34	
III	6	8	39	21	
IV	2	0	1	1	
Underlying cardiac disease (%)					
Ischemic heart disease, leading diagnosis	60	66	58	61	0.652
CABG	21	18	30	25	0.239
PCI	27	44	37	36	0.217
Cardiomyopathy, other	25	26	27	26	0.668
Arrhythmia and conduction defects (%)					
Non permanent atrial fibrillation	15	24	31	25	0.108
Atrial tachycardia	4	14	10	10	0.251
Ventricular fibrillation	27	12	13	16	0.066
Ventricular tachycardia (sustained)	25	34	8	19	0.003^1,2^
Ventricular tachycardia (non-sustained)	21	26	16	20	0.372
AV-block	10	10	28	19	0.008^2^
Medication (%)					
Beta-blocker	91	85	90	89	0.333
Calcium channel blocker	18	11	6	10	0.129
Digoxin	0	2	13	7	0.004^2^
Anti-arrhythmics	25	34	29	29	0.599
of which amiodarone	14	28	25	23	0.187
Implantation type (%)					
New	38	59	39	44	< 0.001^1,2^
Replacement	62	37	33	41	
Upgrade	0	4	28	15	

Significant post-hoc comparisons are indicated as: ^1^ SC-ICD vs CRT-D; ^2^ DC-ICD vs CRT-D

Patients were followed for a mean (± SD) duration of 27.7 (8.6) months, range 0.8 to 44.4 months, with similar durations of follow-up in the three ICD groups (28.1, 27.2, and 27.7 months, respectively). Device programming strategies diverged from recommendations (Figs. 5 and 6 in Appendix) in all patients but one (Table 4 in Appendix); in particular, VT cycle length was programmed longer than recommended in 71.4% of the devices (mean 353 ± 27 and 368 ± 51 ms in primary and secondary prevention, respectively) and the Number of Intervals to Detect (NID) was programmed shorter than recommended in 47.4%. This phenomenon has been observed in all participating centers. Programmed mean SVT limit was consistent with recommendations (256 ± 17 and 259 ± 19 ms in primary and secondary prevention, respectively). Regarding VF cycle length, there was no significant difference between primary and secondary prevention (295 ± 22 and 294 ± 22 ms, respectively) and no significant difference between SC/DC/CRT-D. In all patients features of SST were turned “ON” by default. For further details on programming divergences, refer to the Table 4 in Appendix.

### Device-based management of detected VTAs

**Fig. 2:**
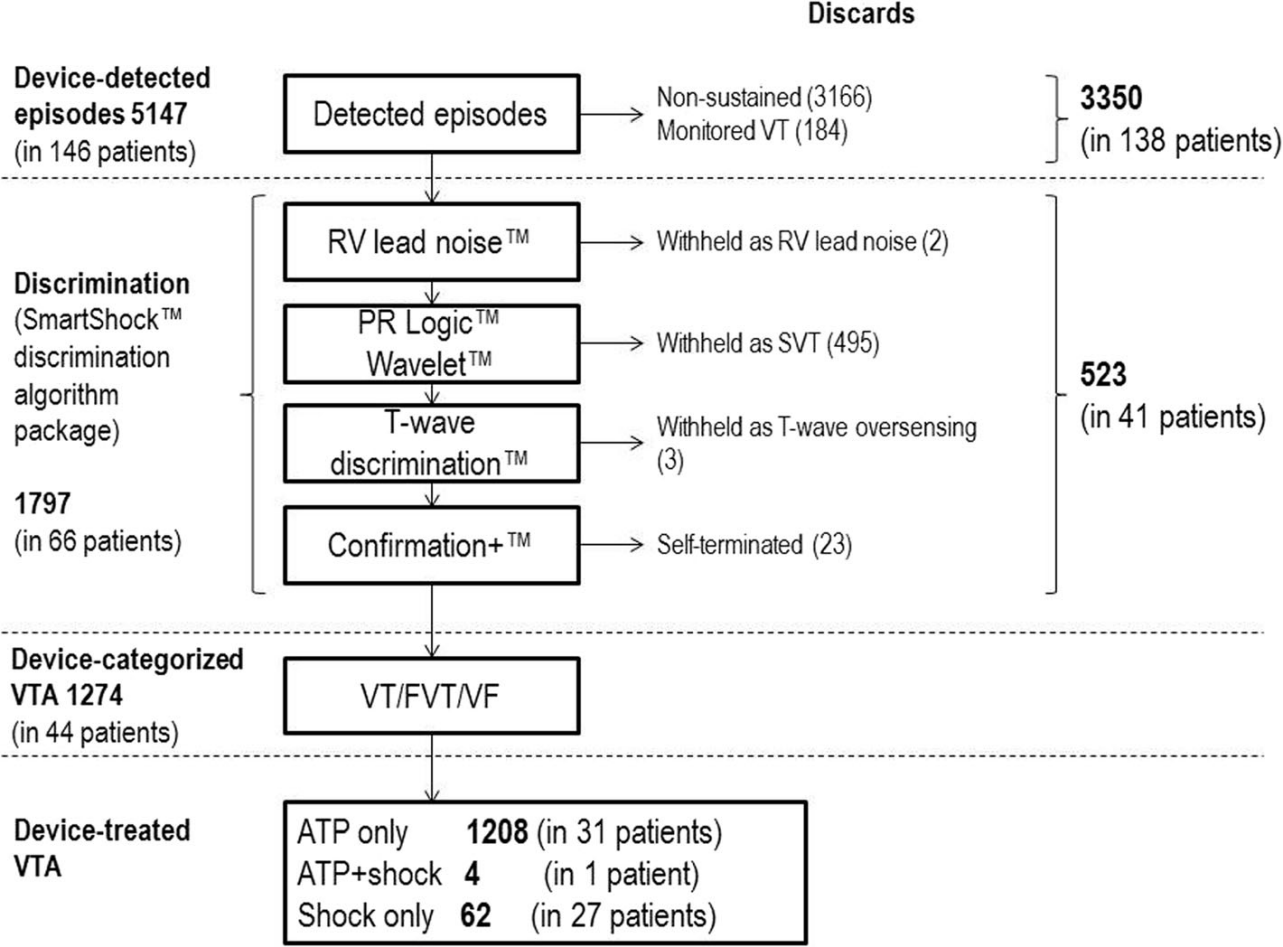
Sequential management of ventricular tachyarrhythmia (VTAs) by ICD devices, from the detection to the electrical therapy of categorized VTAs

During the follow-up period, 5147 episodes were device-detected, of which 1797 (34.9%) were device-categorized as possible VTAs (Fig. 2). Of these, 523 were withheld from therapy delivery by SST (Fig. 2). As shown in Table 2, the remaining 1274 VTA episodes (1161 VT, 16 FVT, 97 VF) resulted in device therapy delivery: 1208 ATP only, 62 shock only, and 4 ATP followed by a rescue shock. The overall device-therapy (ATP ± rescue shock) delivery rate was 2.8 (95% CI 2.7–3.0) per 100 patient-years (3.3 (3.0–3.7) for SC-ICD, 6.6 (6.2–7.1) for DC-ICD, and 0.7 (0.6–0.8) for CRT-D). The ATP success rate was 99.9% (1207 successfully treated episodes of 1208 treated) and the shock success rate was 100% (60 successful shock episodes out of 60 delivered) when excluding the single patient with an electrical storm (one non-successful ATP followed by six non-successful shocks and one successful shock – counted as one episode). A total of seven inappropriate interventions occurred in 5 patients (2.6%) due to noise/artefacts (1 ATP, 1 ATP + shock, 2 shocks), non-sustained VT (1 shock), and SVT (2 shocks).

**Table 2:** Device-based treatment of ventricular tachyarrhythmia episodes, as number of episodes (number of patients) having experienced one or more episode

Delivered therapy (%)	VT (91.1%)	FVT (1.3%)	VF (7.6%)	Total
ATP only (94.8%)	1146 (23)	11 (4)	51 (11)	1208 (31)
SC-ICD	327 (11)	7	22	356
DC-ICD	739 (4)	2	1	742
CRT-D	80 (8)	2	28	110
ATP + shock (0.3%)	0 (0)	0 (0)	4 (1)	4 (1)
SC-ICD	0 (0)	0 (0)	0 (0)	0 (0)
DC-ICD	0 (0)	0 (0)	0 (0)	0 (0)
CRT-D	0 (0)	0 (0)	4 (1)	4 (1)
Shock only (4.9%)	15 (7)	5 (4)	42 (19)	62 (27)
SC-ICD	6 (3)	0 (0)	8 (5)	14 (8)
DC-ICD	1 (1)	2 (1)	9 (5)	12 (7)
CRT-D	8 (5)	3 (2)	25 (9)	36 (12)
Total	1161 (26)	16 (6)	97 (25)	1274 (44)

The risk of experiencing one or more VTAs was higher in patients with a history of VT/VF episodes (Odds Ratio (OR) 2.9, 95% CI 1.4–6.0, *p* = 0.004) and in patients with a secondary prevention indication for ICD implant (OR 2.4, 95% CI 1.3–4.4, *p* = 0.006). Patients treated with a CRT-D were at lower risk of such events (OR 0.5, 95% CI 0.3–0.9, *p* = 0.016). Consistently, delivery of ATP therapy was significantly more likely in patients with a history of VT/VF (OR 3.5, 95%CI 1.5–8.2, *p* = 0.003) or with a secondary prevention indication (OR 3.1, 95%CI 1.4–6.8, *p* = 0.005), and less likely in patients treated with a beta-blocker (OR 0.3, 95% CI 0.1–0.7, *p* = 0.004), with similar findings with regard to rescue shock delivery.

In total, 146 of 196 patients (74.5%) had one or more device-detected VTA during the 2.3 years of observation (Fig. 2). Of these, 44 patients (22.4%) had experienced one or more VTA episodes per patient that resulted in therapy delivery by the device: 26 patients (59.1%) with VT episodes, 6 (13.6%) FVT, and 25 (56.8%) VF (Table 2). Treatment success rates at the patient level were 100%, for both ATP and shock therapies.

### Adjudicated symptomatic events

During the course of the study, 215 symptomatic clinical events suggestive of spontaneous VTAs were reported in 45 patients. Using device recordings, 175 episodes (81.4%, in 39 patients) were adjudicated as appropriate VTA episodes: 156 VT, 7 FVT, and 12 VF were treated by either ATP only (125), ATP followed by rescue shock (13), or shock only (20), and 17 episodes self-terminated before therapy was delivered. The remaining episodes were not-classified (29), inappropriate interventions (7; see above), or short non-sustained ventricular tachycardia episodes (4).

### Mortality

**Fig. 3:**
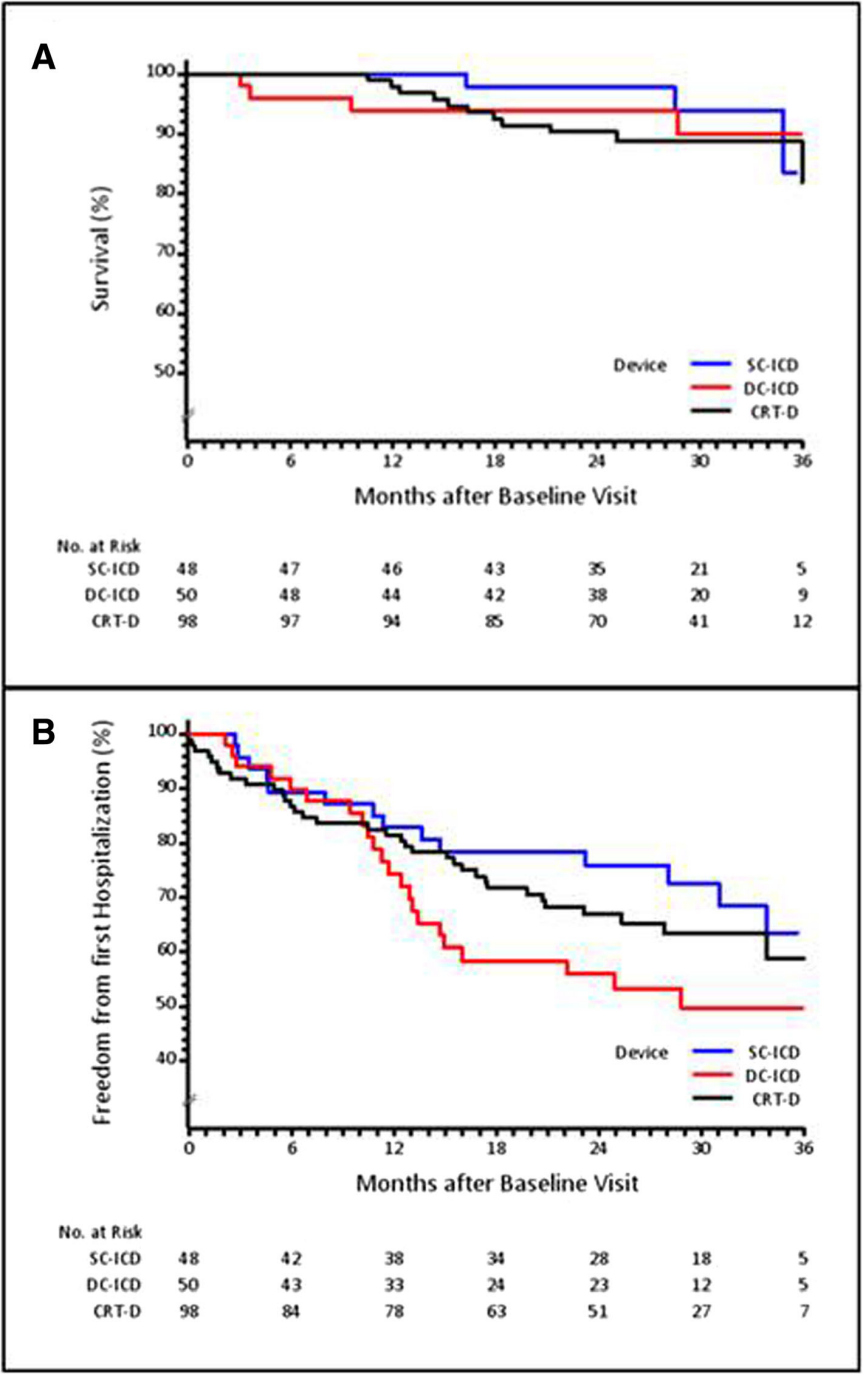
Kaplan-Meier curves for survival (**a**) and freedom of hospitalization (**b**)

Of the 196 patients included in the analysis, 19 (9.7%) died during the 2.3 years of observation, 3 in the SC-ICD group, 4 in the DC-ICD group and 12 in the CRT-D group, corresponding to annual mortality rates of 2.6, 3.5, and 5.3 per 100 patient-years, respectively (Table 3, Fig. 3a). Ten patients died of non-cardiac causes, including 2 of pneumonia, 2 after suicide, and 1 each of cancer, end-stage renal disease, stroke, pulmonary embolism, amyotrophic lateral sclerosis, and one not further specified. Seven patients (all CRT-D patients) died of cardiac causes (4 of worsening heart failure, 2 of acute myocardial infarctions, and 1 of a not shockable recurrent VF). Two patients died of unreported causes. No cases of death were temporally associated with a shock.

**Table 3:** Death and Hospitalization rates per 100 patient-years (95%CI) by cause and device type

	SC-ICD	DC-ICD	CRT-D	Overall
Death	2.6 (0.8–8.3)	3.5 (1.3–9.4)	5.3 (3.0–9.4)	4.2 (2.6–6.6)
Any hospitalization	16.0 (10.1–25.4)	36.1* (26.6–49.1)	28.3 (22.2–36.2)	27.2 (22.8–32.5)
Non-cardiac hospitalization	7.1 (3.6–14.2)	17.6 † (11.4–27.3)	14.2 †† (10.0–20.0)	13.3 (10.3–17.1)
Cardiac hospitalization	8.9 (4.8–16.5)	18.5 ** (12.1–28.4)	14.2 (10.0–20.0)	13.9 (10.9–17.8)
Hospitalization for arrhythmia	1.8 (0.4–7.1)	7.0 ^#^ (3.5–14.1)	5.8 ^##^ (3.3–9.9)	5.1 (3.4–7.7)
Ventricular pacing %	6.9 (0–29.2)	11.3 (0–41.1)	96.8 (92.8–100.0)	/

Mean values with corresponding standard deviation between parenthesis

* *p* = 0.010 vs. SC-ICD

† *p* = 0.03 vs. SC-ICD; †† *p* = 0.045 vs. SC-ICD

** p = 0.04 vs. SC-ICD

^#^ *p* = 0.017 vs. SC-ICD; ^##^ *p* = 0.043 vs. SC-ICD

### Hospitalizations

Overall, 123 hospitalizations of any cause occurred in 72 patients, of which 63 hospitalizations for cardiac reasons (in 47 patients), 23 hospitalizations for arrhythmia (in 14 patients). As shown in Table 3 and Fig. 3b, hospitalization rates per 100 patient-years were significantly higher in the DC-ICD group compared to the SC-ICD group (any cause, non-cardiac and cardiac causes, and arrhythmia). Overall mean length of stay was 14.3 ± 40.4 days (a single patient with long-term hospitalization) with corresponding values of 4.9 ± 4.2, 21.7 ± 68.8, and 12.6 ± 17.1 days for the SC-ICD, DC-ICD and CRT-D groups, respectively. The risk of hospitalization was increased in patients with heart failure NYHA-class III-IV (any hospitalization with Hazard Ratio (HR) 2.4, 95%CI 1.4–4.1, *p* = 0.002 and cardiac hospitalizations with HR 2.9, 95%CI 1.4–6.1, *p* = 0.004) and in patients taking an anti-arrhythmic drug (any hospitalization HR 2.1, 95%CI 1.2–3.5, *p* = 0.006).

### Serious adverse device effects (SADE)

Nine SADE (in 6 patients) were reported, including 5 right ventricular lead dysfunctions, 2 device dysfunctions (1 battery end-of life state and 1 device dislocation), and 2 cases of device pocket infection.

## Discussion

The 4P study reports the characteristics of electrical therapies for ventricular tachyarrhythmic events in a cohort of non-selected, non-trial patients with a class I clinical indication for ICD therapy. Although evidence-based ICD programming strategies were recommended, “patient-specific” programming was preferred with SST features left “ON” in every patient. One or more generally asymptomatic VTAs were detected in 75% of all included patients during 27.7 months of observation. Device-categorized VTAs represented 25% of all device-detected events; SST discrimination capability was highly accurate with a PP value of 99.5%. Ninety-five percent of these episodes were treated by ATP and 5% by a rescue shock with a therapy success rate of 100% at the patient-level. Overall, the present study confirms the results of the PainFree SST study [13] in a real-life clinical setting.

**Fig. 4:**
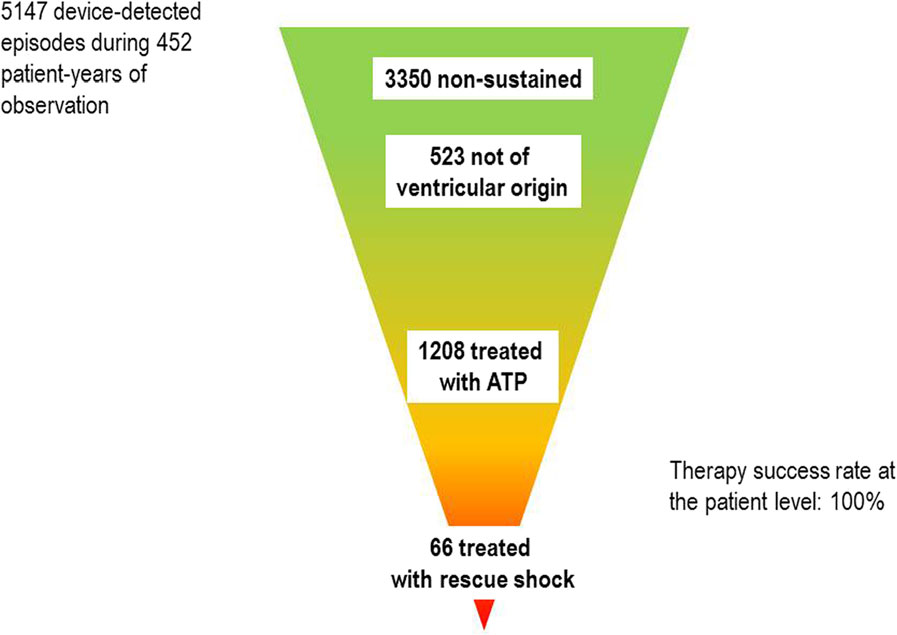
Last generation ICDs are primarily ATP devices with defibrillation backup (rescue shock)

In the present study, device-detected episodes were frequent (5147 in 146/196, 75% patients during 27.7 months of follow-up). One fourth of these (1274) were device-categorized as VTAs and electrical therapy was applied (Fig. 4). Interestingly, although patients were instructed to seek for medical advice in the case of symptoms suggesting underlying arrhythmia, only 45 patients reported 215 such events of which 81% were adjudicated as VTAs. This might suggest that most arrhythmic events and ATP therapies remain asymptomatic. In the recently published results of the Spanish UMBRELLA registry, in which devices preceding SST generation devices were also included, only 5951 VTAs were detected in 605 of 1514 included (40%) patients during 26 months of follow-up with 3353 (56%) categorized as VTAs (56%) [12].

Overall in 4P, 95% of all device-categorized VTAs were effectively treated with ATP and only 5% required a rescue shock. Antitachycardia pacing and shocks restored sinus rhythm in 99.9 and 100.0% of the episodes, respectively. In the UMBRELLA registry, approximately 80% of the VTA episodes were treated with ATP and 20% required a rescue shock [12]. The shock rate in 4P (15 per 100 patient-years) was similar to that published in PainFree SST (16 per 100 patient-years) [13] and in ADVANCE-III (19 and 30 per 100 patient-years in the NID 30/40 and 18/24 treatment groups, respectively) [16]. The ATP rate in 4P was higher than in the two former trials which can at least partially be explained by a shorter than recommended NID in almost 50% of the patients. The latter may have contributed to an increased number of ATP-treatments, consistent with the findings in the ADVANCE-III [16, 17] and PainFree SST [13] trials. Another reason accounting for the high rate of ATP therapies is related to the large proportion of patients who presented a secondary prevention ICD indication and underwent device replacement.

**Fig. 5:**
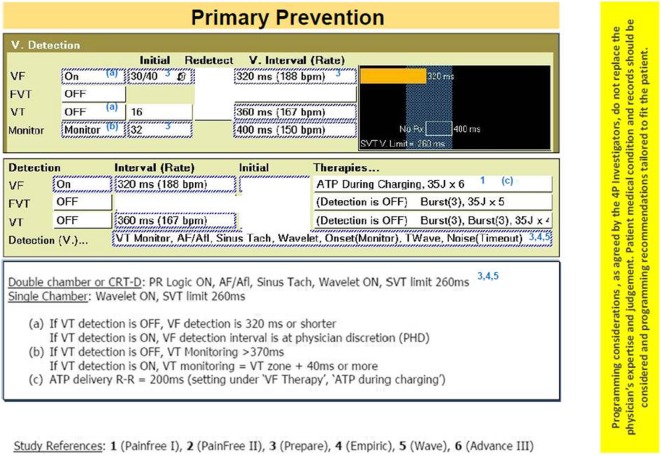
Specific programming information handed out to implanters before trial initiation (primary prevention)

**Fig. 6:**
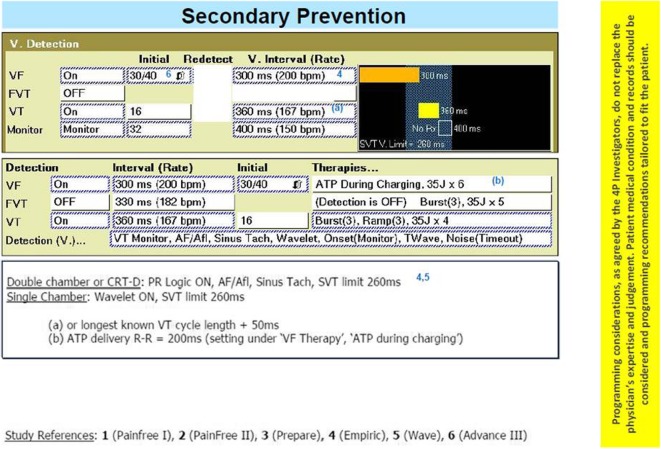
Specific programming information handed out to implanters before trial initiation (secondary prevention)

Worthy of note, despite evidence-based programming recommendations [13, 14, 16], in 4P patient-specific settings were usually preferred and only one ICD was programmed in full compliance with the handed-out recommendations (Figs. 5 and 6 in Appendix). In spite of this, the inappropriate shock rate in 4P (2.6% of the patients during 27.7 months of follow-up) was lower compared to the UMBRELLA registry (5% during 25 months) [12] and the PainFree SST trial (2.8 and 3.7% during 22 months for patients with dual/triple-chamber and single chamber ICDs, respectively) [13]. Moreover, the incidence of inappropriate shocks was much lower than in the pivotal RCTs (10 to 24% of the patients during 20 to 45 months) [18].

While fully acknowledging that individual ICD programming may be required in specific clinical situations, it appears unlikely that virtually all patients included in 4P had characteristics diverging from those of the validation trials. The EMPIRIC trial clearly showed that standardized empiric ICD programming for VT/VF settings was at least as effective as patient-specific programming [3]. Whether similar findings might still be expected with last generation ICDs could obviously not be explored in the context of the 4P study.

Cardiovascular hospitalization rates were low in 4P, representing approximately 50% of the rates published in the ADVANCE III trial [19]. However, in 4P (Table 3 and Fig. 3b), patients implanted with a DC-ICD experienced significantly more hospitalizations (for any cause, for cardiac causes and for arrhythmic causes) and had more VTAs requiring electrical therapy than patients implanted with a SC-ICD. Compared to patients implanted with SC-ICD, DC-ICD patients in 4P were generally older, sicker at baseline, and presented a higher incidence of negative prognostic factors (including cardiovascular disease and arrhythmia history), precluding direct comparison between groups and indirect comparison with other studies.

### Study limitations

The present study has some limitations. First, ICD patients with heart failure disease and left ventricular dysfunction often present with co-existing comorbidities such as diabetes mellitus, metabolic syndrome, and moderate-to-severe renal impairment. These conditions and their therapeutic control over time may have had an important impact on hospitalization rates, mortality rates, as well as ventricular arrhythmic burden [20–23]. Furthermore, the identification of patients at higher risk by integrating echocardiographic evaluations of cardiac function and left ventricular dimensions with neuroendocrinal and inflammatory parameters could certainly have been relevant. However, neither detailed echocardiographic evaluation nor sampling of particular hematologic markers, were defined by the study protocol. Second, patients with persistent atrial fibrillation were not included. This may have contributed to the low rate of inappropriate shocks. However, 25% of the included patients had a history of non-persistent atrial fibrillation. Third, the comparisons between the different ICD models should be considered exploratory as the study was not designed for this purpose. On the other hand, in a context of widely available strong evidence of the benefits and risks of ICD therapy, 4P offers unique real-world insights into the patient profiles, arrhythmic events and electrical therapies as they occur in daily routine and confirms that results achieved in clinical outcome trials are reproducible in conditions of daily practice. Fourth, patients included in 4P were followed during only 2 years with 44% of them receiving a new implant. Long term studies over 11 years which included patients who underwent multiple ICD device replacements suggest that the proportion of patients experiencing appropriate and inappropriate shocks may increase over time [24]. Thus, the low rates of appropriate and inappropriate shocks observed in 4P may be explained, at least in part, by the comparatively short duration of observation.

## Conclusion

In conclusion, in a daily practice setting of ICD patients with a class I indication for an ICD, device-detected, −categorized and –treated episodes were frequent. Almost one patient out of every four experienced one or more potentially lethal ventricular tachyarrhythmia in the course of a two-year follow-up and benefitted from life-saving electrical therapy, mostly through effective ATP delivery. Overall, the present real-life results confirm the robustness SST for the detection, categorization, and treatment of ventricular arrhythmias to limit the overall incidence of ICD shocks. More studies would be desirable to confirm the effect of SST on the long-term technical and medical outcomes of ICD patients.

## Data Availability

The datasets used and/or analysed during the current study are available from the corresponding author on reasonable request.

## Appendix

**Table 4:** Details on the ICD programming divergences from the recommended settings (presented as Figures 5 and 6 in Appendix)

	TOTAL (*n* = 185)	SC-ICD (*n* = 44)	DC-ICD (*n* = 48)	CRT-D (*n* = 93)
Primary prevention (pts)	121	19	25	77
FVT detection- ON [n, (%)]	19 (10.2)	1 (2.3)	4 (8.3)	14 (15.1%)
VT detection- ON [*n*, (%)]	90 (74.4)	15 (78.9)	18 (72.0)	57 (64.0)
PR Logic/ Wavelet- OFF or Monitor [*n*, (%)]	18 (14.9)	3 (15.8)	2 (8.0)	13 (16.9)
Onset ON or OFF [*n*, (%)]	49 (40.5)	12 (53.1)	9 (36.0)	28 (36.4)
T-Wave- OFF [*n*, (%)]	6 (5)	3 (15.8)	0	3 (3.9)
RV-Lead Noise [*n*, (%)]	3 (2.5)	2 (10.5)	0	1 (1.3)
ATP before charging	8 (6.6)	1 (5.3)	2 (8.0)	5 (6.5)
FVT- ATP & shocks	121 (100)	19 (100)	25 (100)	77 (100)
VT- ATP & shocks	121 (100)	19 (100)	25 (100)	77 (100)
Secondary prevention (pts)	64	25	23	16
FVT detection- ON [*n*, (%)]	19 (29.7)	8 (32.0)	9 (39.1)	2 (12.6)
VT detection- OFF [*n*, (%)]	11 (17.2)	6 (24.0)	5 (21.7)	0
PR Logic/ Wavelet- OFF or Monitor [*n*, (%)]	4 (6.3)	3 (12.0)	0	1 (6.3)
Onset ON or OFF [*n*, (%)]	32 (50.0)	10 (40.0)	14 (60.8)	8 (50.0)
ATP before charging	8 (12.5)	3 (12.0)	2 (8.7)	3 (18.8)
FVT- ATP & shocks	121 (100)	19 (100)	25 (100)	77 (100)

**Table 5:** Classification of 176 symptomatic VTA episodes in 39 patients

	VT (*n* = 22)	FVT (*n* = 10)	VF (*n* = 7)	Total
ATP	125	0	0	125
ATP and shock	11	1	1	13
Shock only	11	1	9	21
Self-terminating	10	5	2	17
Total episodes	157	7	12	176

## Abbreviations

ATP: Anti-tachycardia pacing
CRT-D: Biventricular implantable defibrillator
DC: Dual-chamber
FVT: Fast ventricular tachycardia detection window
ICD: Implantable cardioverter defibrillator
NID: Number of intervals to detect
OR: Odds ratio
RCTs: Randomized controlled trials
SADE: Severe device-related adverse events
SC: Single-chamber
SST: SmartShock™ technology
SVT: Supraventricular tachycardia
VF: Ventricular fibrillation detection window
VT: Ventricular tachycardia detection window
VTAs: Ventricular tachyarrhythmia
